# Treatment of Alveolar Abscess by the Jennings Method

**Published:** 1887-09

**Authors:** 


### The Treatment of Alveolar Abscess by the Jennings' Method.

The filling of roots of teeth with gutta percha dissolved in chloroform is not new. It has been in quite general practice for eight or ten years. But it would seem by the remarks that were made when Dr. Jennings read his paper before this Society, that some at least took it for granted that he was filling roots and nothing more. Although he has given his method in detail, but few seemingly have grasped it in its entirety.

Let us at the outset bear this in mind—that by this mode of treatment is meant injecting root, sack, and canal to its outlet with gutta percha dissolved in chloroform. In the first place free the root, sack, and outlet of its effete matter. It makes but little difference with what you do this—if nothing more than warm water, be sure to do it thoroughly—place it in as favorable condition as possible for nature to repair the damage done. Nature's tendency being to reproduce, why not fill the offending root and sack as soon as it is thoroughly renovated—which can be done at one sitting better than a dozen—and if as has been suggested there be caries or necrosis let it be treated externally as such. You will find the abscess was wholly obliterated by the injection. It being the opinion of the writer that the diseased bony structure is as much benefited by the injection as are the soft parts, but being slower to repair do not respond to the stimulating influences as quickly.

That the presence of this solution is stimulating and beneficial we hope to prove before we are done. At the reading of Dr. Jennings' paper many objections were raised to forcing this solution beyond the apex of the root and leaving it there, without giving any valid reasons for such objections. We will try and clear away this cloud and make the value of this treatment so evident that it will succeed all others. We will make this assertion: There is no case of alveolar abscess—no matter of how long standing—if the periosteal covering of the root is intact that will not yield to this treatment. The periosteum need not be entire; simply sufficient to give it attachment.

Before proceeding farther let us look briefly at the old method of treating these cases and see how little logic it contains. We mean the pumping of carbolic, sulphuric acid, or what not, through the root and opening. One treatment of this kind might not be out of place if the root and sack be directly filled with the chloro-percha solution. But look you what follows. After the first treatment the root is loosly packed with cotton and left open to the deleterious influences of the air, saliva, and its impurities. After one or two day's wasting the same process is repeated—forcing these acids through the channel nature has been trying to close with new tissue, and in many cases destroying what little advance that has been made toward restoration. Thus it is repeated at intervals for weeks, perhaps months, and the only reason we can see for Nature's coming out ahead in the race is because she is more persistent in her efforts to repair, than is the dentist in his destruction. In many instances teeth of this class have been doctored out of the mouth.

Now let us inquire why this treatment as introduced by Dr. Jennings cures, and what becomes of the solution that passes beyond the apex of the root. That it cures is a demonstrated fact—cure is stamped upon its face and in the manner in which it is used. Gutta percha is inert and is only useful as a stopping or plug, and the plug plays no small part in the cure as we will plainly see. In dressing wounds of all kinds it is desirable to bring the parts so completely in contact that there are no pockets in which pus may accumulate. This not being a wound or simply destruction of soft parts—bony structure as well being involved—we can not bring the parts together and thus exclude pus formation, but we fill with a solution that closes the pocket and stimulates nature to effect a cure.

We will next split up the chloroform and see if she does us any good. We have chlorine, an element, and alcohol, a compound of carbon, hydrogen, and oxygen. What are the medical properties of chlorine? It is a deodorizer, a disinfectant and will destroy diseased germs—surely a very fit substance to use in alveolar abscess. Alcohol, antiseptic, destroys germs and stimulates tissues to more healthful action—just the things to introduce into a cavity reeking with corruption and diseased germs. What more could we ask for—and all of this sent directly to the seat of trouble and left there to assist nature in her work. If you please, we have in this solution an antiseptic surgical dressing.

The last item for consideration next claims our attention, viz: What becomes of the gutta percha left in the sack and canal after the medical properties of the solution have disappeared? The opportunity to examine a skull thus treated will rarely present itself, therefore, much of this must be based upon theory. It has been said that it becomes encisted or encapsuled, hence, inert and non-irritating. This may be so. Thus far we have not been able to find what nature elaborates to absorb or break up bodies she may wish to remove. If the contents of the sack and canal are like some we have seen coming from the fistulous opening curdy and finely granular, it might be taken up in the circulation and cast off as effete matter. It might be taken up in this finely divided state—there being no absorption or solution, simply division—as there is in the bony system when the inter-cellular tissue is dissolved out, leaving the chalky lime which is taken up by the circulatory system as lime. In fact we do not know as bony absorption means anything else—it having never been proven that lime is dissolved. May not this be the case with many foreign bodies such as sponge, ivory, cat gut, etc., introduced into the system by surgeons and left there to take care of themselves?

We do not know what nature may do to dissolve bone or any other material, but the supposition of the eminent Dr. Billroth is that in the process of inflammatory new formation, lactic acid is evolved which converts the lime into soluable lactate of lime. If this should be the case the lactic acid will help us get rid of our surplus gutta percha. We find that lactic acid acts upon the solution of gutta percha and chloroform to such an extent as to render it granular after being in it a few hours at the temperature of 850°. As there is no possible way of demonstrating the truth of these theories we will have to content ourselves with the fact that the method of treatment is a success, and that the gutta percha, no matter what becomes of it, has never given us any after trouble.

